# Differentially Expressed Genes Identification of Kohlrabi Seedlings (*Brassica oleracea* var. *caulorapa* L.) under Polyethylene Glycol Osmotic Stress and *AP2/ERF* Transcription Factor Family Analysis

**DOI:** 10.3390/plants13081167

**Published:** 2024-04-22

**Authors:** Shuanling Bian, Mengliang Zhao, Huijuan Zhang, Yanjing Ren

**Affiliations:** 1 State Key Laboratory of Plateau Ecology and Agriculture, Laboratory of Research and Utilization of Germplasm Resources in Qinghai-Tibet Plateau, Qinghai University, Xining 810016, China; bianshuanling2024@163.com (S.B.); zhangzhang1046@163.com (H.Z.); 2 Key Laboratory of Germplasm Resources Protection and Genetic Improvement of the Qinghai-Tibet Plateau in Ministry of Agriculture and Rural, Xining 810016, China

**Keywords:** kohlrabi (*Brassica oleracea* L. var. *caulorapa* L.), polyethylene glycol osmotic stress, differentially expressed genes, *AP2*/*ERF* transcription factor, gene expression

## Abstract

Osmotic stress is a condition in which plants do not get enough water due to changes in environmental factors. Plant response to osmotic stress is a complex process involving the interaction of different stress-sensitive mechanisms. Differentially expressed genes and response mechanisms of kohlrabi have not been reported under osmotic stress. A total of 196,642 unigenes and 33,040 differentially expressed unigenes were identified in kohlrabi seedlings under polyethylene glycol osmotic stress. *AP2*/*ERF*, *NAC* and eight other transcription factor family members with a high degree of interaction with CAT and SOD antioxidant enzyme activity were identified. Subsequently, 151 *AP2*/*ERF* genes were identified and analyzed. Twelve conserved motifs were searched and all *AP2*/*ERF* genes were clustered into four groups. A total of 149 *AP2*/*ERF* genes were randomly distributed on the chromosome, and relative expression level analysis showed that *BocAP2*/*ERF* genes of kohlrabi have obvious specificity in different tissues. This study lays a foundation for explaining the osmotic stress resistance mechanism of kohlrabi and provides a theoretical basis for the functional analysis of *BocAP2*/*ERF* transcription factor family members.

## 1. Introduction

Kohlrabi (*Brassica oleracea* L. var. *caulorapa* L.) is a variety of cabbage (*Brassica oleracea*) that belongs to the Cruciferae family (Brassicaceae) [1], and is widely cultivated in Europe, the US, Canada and Asia [2]. The swollen stem at the base of the plant is mainly consumed by humans as food [3,4]. It is a valuable source of nutrients, mainly containing carotenoids, glucosinolates, and phenylpropanoids [5]. In addition, potential antidiabetic, anti-inflammatory, and antioxidant properties and anticancer effects have been found in kohlrabi [6].

Osmotic stress occurs when the osmotic pressure in a plant is lower than the environmental osmotic pressure, and thus the plant cannot absorb or even lose water. This leads to a physiological drought caused by water deficit and ultimately affects plant growth and development. Water deficit is an important abiotic stress factor that limits agricultural crop production worldwide [7,8,9,10]. Water deficit affects plant growth, development, and productivity at any stage [11]. Due to varied species, different drought stress severity, duration, and the timing of water deficit occurrence, physiological and gene network responses to water deficit are complex [10,12]. Therefore, it is of great significance to study response mechanisms to physiological drought caused by osmotic stress and excavate differentially expressed genes of different crops. Relying on transcriptome sequencing approach, a large number of differentially expressed genes (DEGs) in response to Polyethylene Glycol (PEG) osmotic stress were identified [13,14]. In root and tuber crops, Karanja et al. [15] reported that the *RsERF045*, *RsERF104* and *RsERF184* genes were highly responsive to PEG in radish. Öztürk Gökçe et al. [16] described MYB-48 transcription factor, F-box protein, ferric reduction oxidase, and ABA-induced somatic embryo gene expression upregulation under water deficit. Zhao et al. [10] reported that *NAC*, *MYB*, *WRKY*, *homeobox-leucine zipper* (*HD-ZIP*) and *basic leucine-zipper* (*bZIP*) were closely related to the response to PEG osmotic stress in Jerusalem artichoke. In potato, Charfeddine et al. [17] revealed that overexpression of *StERF94* improved the tolerance of transgenic plants to drought, heat, and combined stresses. However, the DEGs and responding mechanism of kohlrabi under water deficit induced by osmotic stress have not been reported.

APETALA2/ethylene responsive factor (*AP2*/*ERF*) is one of the largest transcription factor families in plants, with a typical feature of one or two AP2 DNA-binding conserved domains [18,19]. *AP2*/*ERF* transcription factor family members are divided into AP2, ERF, and RAV subfamilies based on the number and type of conserved domains [20]. The AP2 subfamily members contain two AP2 domains, while the ERF subfamily members contain an AP2 domain, and the RAV subfamily members contain one AP2 domain and one B3 domain [21,22,23]. Research on the AP2/ERF family has been conducted in a variety of plants, including rhododendron [24], oily persimmon [25], maize [26], sand pear [27], tomato [28], *Juglans mandshurica* [29], ramie [30], and so on. The identification and analysis of *AP2*/*ERF* family members lays the foundation for clarifying the function of the *AP2*/*ERF* gene.

In this study, DEGs under osmotic stress and the *AP2*/*ERF* transcription factor family in kohlrabi are reported for the first time. This study lays a foundation for explaining the osmotic stress resistance mechanism of kohlrabi and provides a theoretical basis for the function analysis of *BocAP2/ERF* transcription factor family members.

## 2. Results

### 2.1. Changes in SOD Activity, CAT Activity, and Proline Content under Osmotic Stress

**Figure 1:**
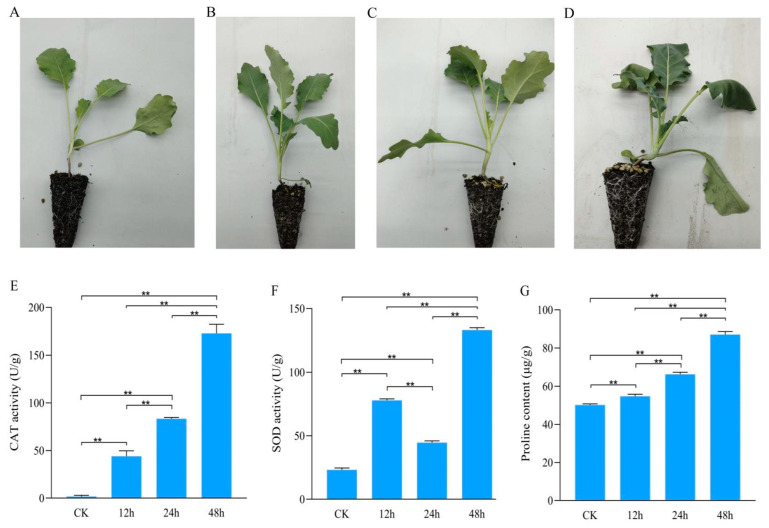
Phenotypes and biochemical indicators of kohlrabi seedlings under PEG6000 osmotic stress treatment. (**A**–**D**) Phenotypes of kohlrabi seedlings under PEG6000 osmotic stress treatment at 0 h, 12 h, 24 h and 48 h; (**E**) catalase activity of kohlrabi seedlings under osmotic stress at 0 h, 12 h, 24 h and 48 h; (**F**) superoxide dismutase activity of kohlrabi seedlings under osmotic stress at 0 h, 12 h, 24 h and 48 h; (**G**) proline content of kohlrabi seedlings under osmotic stress at 0 h, 12 h, 24 h and 48 h. ** represents significantly difference when p value was 0.01.

To study the physiology and biochemical effect of osmotic stress treatment on kohlrabi seedlings, kohlrabi seedlings were treated with PEG-6000 and phenotypes observed and photographed (Figure 1A–D). Kohlrabi seedling leaves were dehydrated at 24 h and wilted at 48 h after PEG-6000 treatment. According to the phenotype changes, seedlings leaves were sampled at 0 h (untreated, CK) and 12 h, 24 h, and 48 h after treatment and each sample contains three biological replicates.

Biochemical indicators, including CAT activity, SOD activity and proline content, were detected. With increasing treatment time, the activity of CAT had significantly increased from 1.68 ± 1.33 U/g FW at CK to 172.89 ± 9.55 U/g FW at 48 h after treatment (Figure 1E). The change trend of SOD activity varied from that of CAT activity with increasing treatment time. SOD activity was significantly stronger after treatment than that in control and significantly weaker at 24 h (44.65 ± 1.32 U/g FW) after treatment than those at 12 h (77.87 ± 1.67 U/g FW) and 48 h (133.16 ± 1.76 U/g FW) after treatment (Figure 1F). Proline content was significantly increased with increasing treatment time: 50.18 ± 0.61 μg/g FW at CK and 87.02 ± 1.61 μg/g FW at 48 h after treatment (Figure 1G).

### 2.2. Unigene Annotation and Differentially Expressed Gene Identification under Osmotic Stress

Twelve RNA-sequence libraries were built and used for analyzing DEGs in kohlrabi seedlings under PEG-6000 osmotic stress. A total of 45,262,254 – 53,167,538 raw reads and an average of 46,526,798 clean reads with 6.98 Gb clean base were generated. Data quality analysis showed that the average Q30 was 94.40% and the average GC content 47.15% (Table S1). After Trinity splicing, 204,346 transcripts were generated with an average length of 1304 bp, with N50 length of 1834 bp and N90 length of 664 bp. Then, upon Corset hierarchical clustering, 196,642 unigenes were obtained with an average length of 1345 bp, an N50 length of 1844 bp, and an N90 of 689 bp.

Transcript and unigene length analysis showed that 38,951 transcripts (19.06%) and 38,951 unigenes (19.80%) were more than 2000 bp in length, while 22,900 transcripts (11.21%) and 15,804 unigenes (8.04%) were less than 500 bp in length (Figure S1A). Unigene annotation revealed 134,011 (68.15%), 173,759 (88.36%), 144,868 (73.67%), 103,662 (52.72%), 123,671 (62.89%), 125,704 (63.93%) and 175,825 (89.41%) unigenes were annotated in the Kyoto Encyclopedia of Genes and Genomes (KEGG), NCBI non-redundant (NR) protein sequence, Gene Ontology (GO), Eukaryotic Orthologous Groups (KOG), Protein Family (Pfam), Swiss-Prot and Trembl databases, respectively. A total of 177,301 unigenes (90.16%) were annotated in at least one database and all unigenes were annotated (Table 1). Alignment with the NR database showed that the species with the most unigene hits (72,081, 41.48%) was *Brassica napus*, followed by *Brassica oleracra* var. *oleracra* (66,448, 38.24%) (Figure S1B).

**Figure 2:**
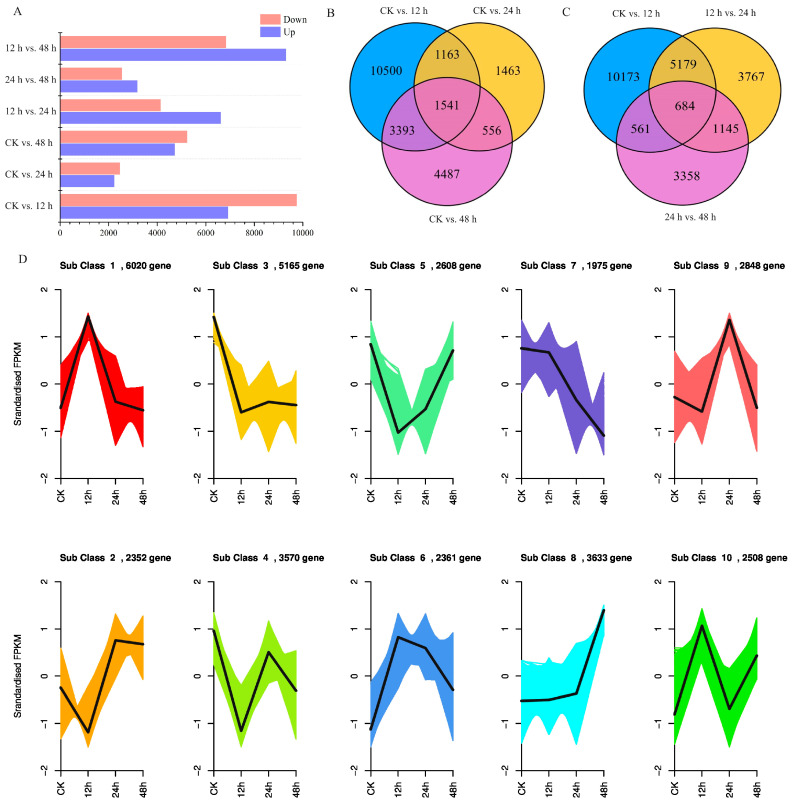
Differentially expressed unigene analysis of kohlrabi seedlings under PEG-6000 osmotic stress treatment. (**A**) Differentially expressed unigenes in different pairwise comparisons between control and different treatment times; (**B**) Venn diagram of differentially expressed unigene numbers in pairwise comparisons between control and treatment of CK vs. 12 h, CK vs. 24 h, and CK vs. 48 h; (**C**) Venn diagram of differentially expressed unigene numbers in pairwise comparisons of treatment of CK vs. 12 h, 12 h vs. 24 h, and 24 h vs. 48 h; (**D**) Trend analysis of differentially expressed unigenes under PEG6000 osmotic stress from 0 h to 48 h.

Based on the FPKM value of each unigene in kohlrabi seedling leaves under PEG-6000 osmotic stress, a total of 33,040 DEGs were identified (Table S2). Subsequently, six pairwise comparisons of DEGs at CK vs. 12 h, CK vs. 24 h, CK vs. 48 h, 12 h vs. 24 h, and 24 h vs. 48 h were calculated. Overall, 16,597 (6924 upregulated at 12 h and 9673 downregulated), 4723 (2246 upregulated at 24 h and 2477 downregulated), and 9977 DEGs (4734 upregulated at 48 h and 5243 downregulated) were identified in the comparisons of CK vs. 12 h, CK vs. 24 h and CK vs. 48 h (Figure 2A). Comprehensive analysis of these three pairwise comparisons revealed a total of 1541 common DEGs (Figure 2B, Table S3). In the pairwise comparisons of 12 h vs. 24 h and 24 h vs. 48 h, 10,775 DEGs (6630 upregulated at 24 h and 4145 downregulated), 5748 DEGs (3190 upregulated at 48 h and 2558 downregulated) were identified and three pairwise comparisons (CK vs. 12 h, 12 h vs. 24 h and 24 h vs. 48 h) showed 684 common DEGs (Figure 2C, Table S4).

**Figure 3:**
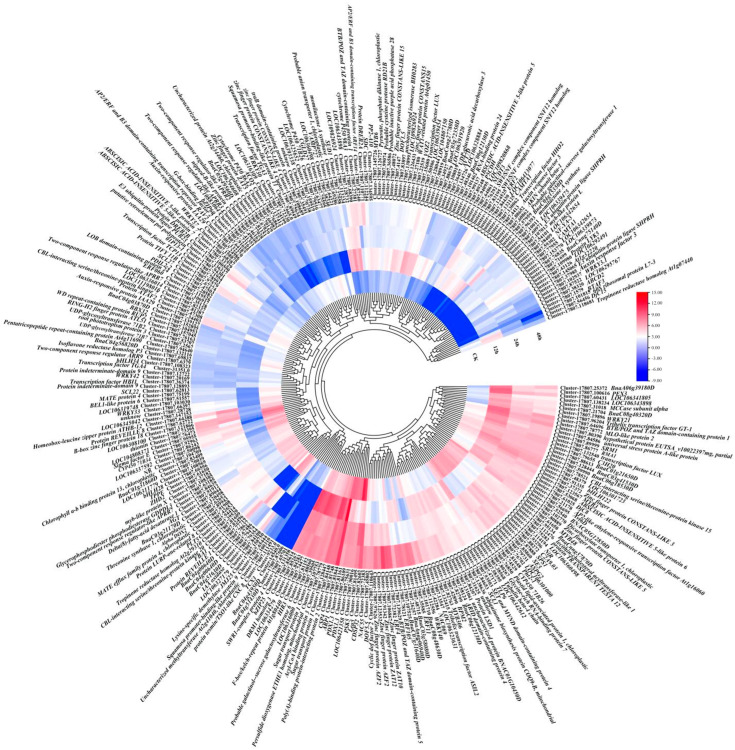
Heatmap of 282 DEGs normalized using log_2_FPKM in leaves of kohlrabi seedlings under PEG-6000 osmotic stress.

Trend analysis was also performed, and results showed that all DEGs were divided into 10 subclasses, in which subclass 7 showed a downward trend and subclass 8 showed an upward trend. A total of 2383 unigenes and 2583 unigenes were clustered separately (Figure 2D, Table S5). To further select the DEGs related to osmotic stress, common DEGs in the pairwise comparisons of CK vs. 12 h, CK vs. 24 h and CK vs. 48 h and pairwise comparisons of CK vs. 12 h, 12 h vs. 24 h and 24 h vs. 48 h were identified. A total of 282 DEGs were screened, and a heatmap of mRNA accumulation is shown in Figure 3.

### 2.3. Validation of the Transcriptomic Data

**Figure 4:**
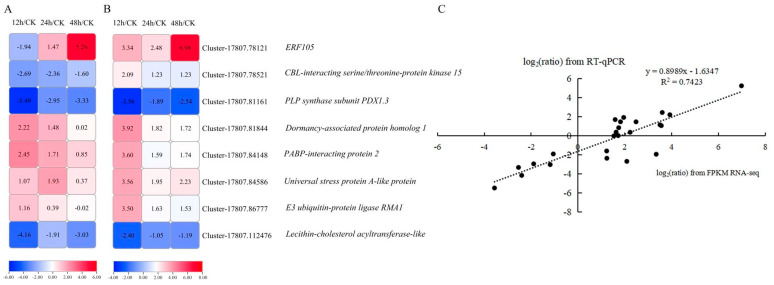
Linear regression analysis of eight DEGs between RNA-sequencing data and RT-qPCR data. (**A**) Expression level heatmap of eight DEGs using RT-qPCR. (**B**) RNA expression profile heatmap of eight DEGs using RNA-seq. (**C**) Linear regression correlation analysis between RNA-seq data and RT-qPCR data of the expression levels of eight DEGs.

To verify the reliability of the transcriptome data, eight randomly selected DEGs were used to analyze expression levels in kohlrabi seedling leaves under PEG-6000 osmotic stress of CK and treatment for 12 h, 24 h, and 48 h by RT-qPCR, with three replicates for each. The qRT-PCR experiment primers are listed in Table S6. The log_2_(ratio) of RT-qPCR and log_2_(ratio) of RNA-seq were analyzed at 12 h/CK, 24 h/CK and 48 h/CK (Figure 4A,B). Despite there existing some tiny differences between RT-qPCR data and the transcriptomic data, the expression trends in these genes were the same (Figure 4C). A significant positive correlation (r^2^ = 0.7423) of the fold change in gene expression rate between RT-qPCR and transcriptome data based on the linear regression analysis results was found, which indicates that the transcriptome data were reliable and valid.

### 2.4. Weighted Gene Co-Expression Network Analysis between Biochemical Indicators and DEGs

**Figure 5:**
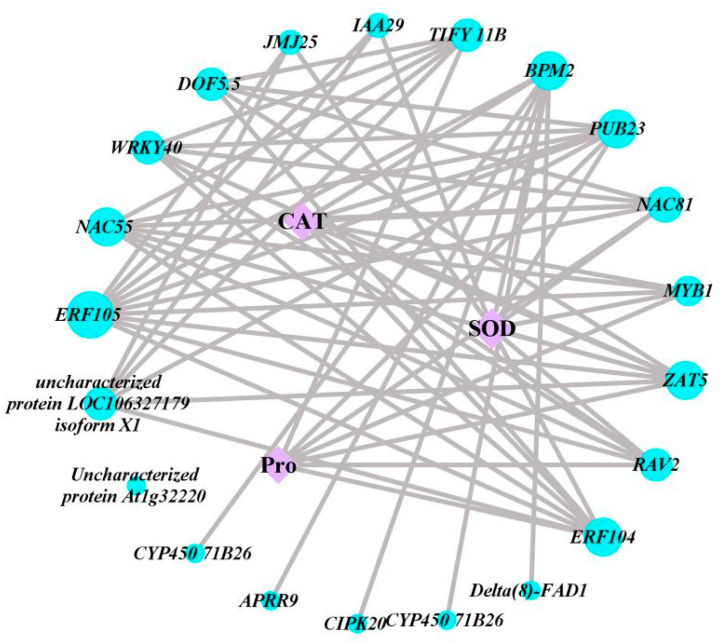
Weighted gene co-expression network map between DEGs and CAT activity, SOD activity and proline content. Blue circles indicate differentially expressed genes; purple squares indicate CAT, SOD and proline.

To explore the key DEGs involved in CAT activity, SOD activity and proline biosynthesis in kohlrabi seedlings under PEG-6000 osmotic stress, the interaction regulatory network between biochemical indicators and DEGs was analyzed by weighted gene correlations. Twenty-one DEGs showed high correlation with CAT activity, SOD activity and proline content, including *AP2*/*ERF* transcription factor family genes, *NAC* transcription factor family genes and eight other transcription factor family genes (Figure 5). The results showed that these genes may adapt to the effects of osmotic stress by promoting or inhibiting CAT activity, SOD activity and proline content.

### 2.5. AP2/ERF Transcription Factor Family Member Identification and Chromosomal Localization

**Figure 6:**
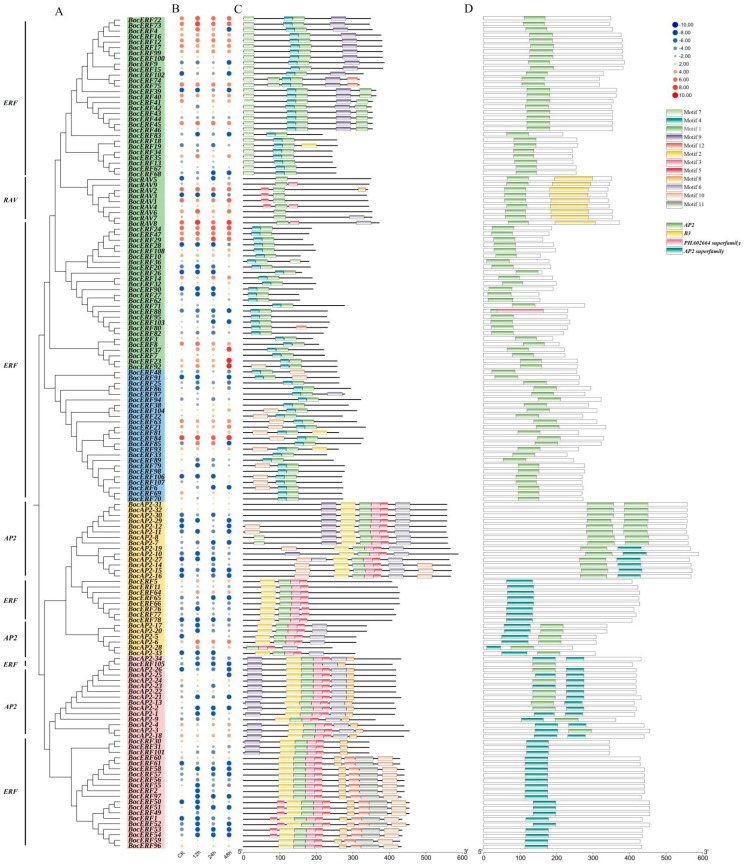
Evolutionary relationships, expression analysis, conserved protein motifs and domains of the 151 *BocAP2*/*ERF*s. (**A**) Phylogenetic relationships were constructed according to amino acid sequences by MEGA11. (**B**) Expression profile of *BocAP2*/*ERF* genes in kohlrabi using RNA sequencing during osmotic stress. (**C**) Distribution of 12 motifs. (**D**) Distribution of AP2 domain, B3 domain, PHA02664 superfamily domain and AP2 superfamily domain.

Considering the high correlation of AP2/ERF transcription factor family genes with CAT activity, SOD activity and proline content, we performed AP2/ERF transcription factor family analysis in kohlrabi. A total of 151 *BocAP2*/*ERF* transcription factors were identified based on a batch CD search of NCBI conserved domain database for the Brassiceae genome (Table S7). The length of BocAP2/ERF proteins ranged from 151 to 588 amino acids (aa) and the molecular weight (MW) ranged from 16,380 Da to 65,563 Da. Physicochemical property analysis showed that the theoretical isoelectric point (pI) ranged from 4.62 to 10.00. Conserved domains analysis showed that 151 members were classified into three subfamilies, including 34 AP2 subfamily genes (two AP2 domains), 108 ERF subfamily genes (one AP2 domain), and 9 RAV subfamily genes (one AP2 and an extra B3 domain) (Figure 6A).

**Figure 7:**
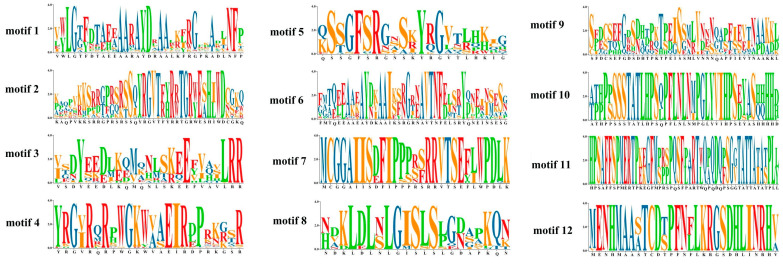
Twelve differently conserved motifs of *BocAP2*/*ERF*s.

The expression profiles of these 151 *BocAP2*/*ERF* genes were also analyzed and showed in Figure 6B. A total of 27 genes showed significant changes under osmotic stress, of which 11 (*BocERF26*, *BocERF28*, *BocERF78*, *BocERF90*, *BocERF104*, *BocERF106*, *BocERF107*, *BocERF108*, *BocAP2-27*, *BocAP2-33*, *BocRAV3*) were upregulated and 16 (*BocERF6*, *BocERF18*, *BocERF44*, *BocERF51*, *BocERF53*, *BocERF54*, *BocERF58*, *BocERF68*, *BocERF74*, *BocERF88*, *BocERF97*, *BocERF100*, *BocERF103*, *BocERF105*, *BocAP2-2*, *BocAP2-7*, *BocAP2-22*) were downregulated. A total of 12 different conserved motifs were searched by MEME (Figure 6C and Figure 7). Motif 1 was present in all BocAP2/ERF members, while motif 5 and 6 were present in the AP2 subfamily, which could be important elements in distinguishing from other subfamilies. No unique motif were found in the RAV or ERF subfamily (Figure 7). The prediction of the conserved structural domains of the 151 BocAP2/ERF proteins revealed that all BocAP2/ERF proteins had highly conserved structural domains. The distribution of the AP2 domain, B3 domain, PHA02664 superfamily domain and AP2 superfamily domain are showed in Figure 6D.

**Figure 8:**
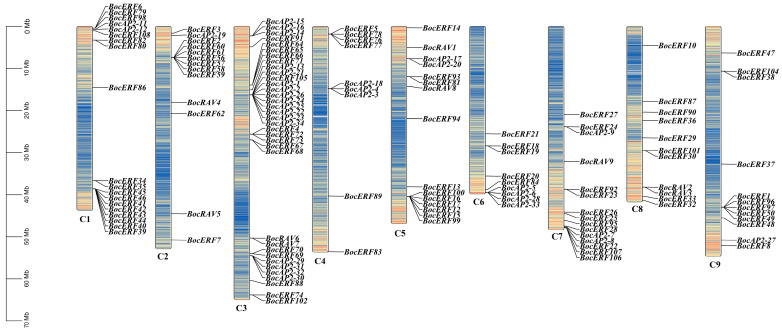
Chromosomal locations of the *BocAP2*/*ERF* genes. The *BocAP2*/*ERF* genes were located on 9 chromosomes, representing gene positions by proportion. Note: C1–C9 indicates 9 chromosomes. Scale bar on the left indicates the chromosome lengths (Mb).

The chromosomal localization results showed that 149 *AP2*/*ERF* genes were randomly distributed on the chromosome (Chr) of kohlrabi and 2 genes were not localized on the chromosome (Figure 8). Chromosome 3 contained 37 genes, while chromosome 4 contained only 9 genes. Chromosome 1, 3, 5, 7 and 9 contained a substantial number of *BocAP2*/*ERF* genes. There was no obvious relationship between chromosome length and the distribution number of *BocAP2*/*ERF* genes. This result revealed an irregular distribution of *BocAP2*/*ERF* genes on the chromosomes.

### 2.6. Evolutionary Analysis of BocAP2/ERF Family Genes

**Figure 9:**
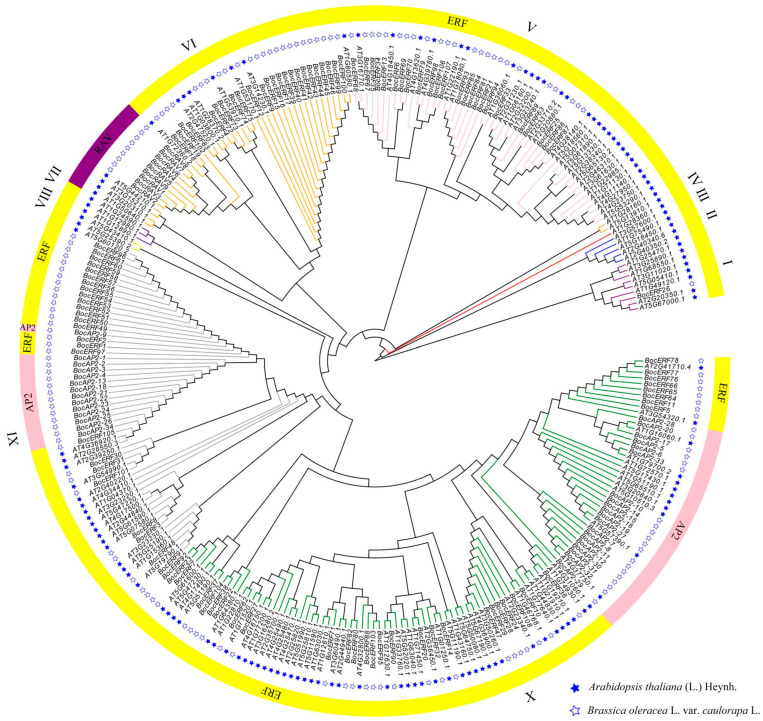
Evolutionary tree of *BocAP2/ERF* family genes in kohlrabi and Arabidopsis.

The evolutionary relationship of 151 *BocAP2*/*ERF* genes from kohlrabi and 136 *AtAP2*/*ERF*s from *Arabidopsis* was constructed and is shown in Figure 9. All the AP2/ERF genes were clustered into 10 groups, named I–X. Among these, the RAV subfamily were distributed into group VI and the AP2 subfamily into group IX and group X. All genes of group II, group III, group IV, group VII and group VIII were obtained from *Arabidopsis*. One *BocERF* subfamily gene, 24 *BocERF* subfamily genes, 24 *BocERF* subfamily genes and 9 *BocRAV* subfamily genes, 25 *BocERF* subfamily genes and 14 *BocAP2* subfamily genes, and 34 *BocERF* subfamily genes and 20 *BocAP2* subfamily genes were clustered in group I, group V, group VI, group IX and Group X, separately.

### 2.7. Expression Analysis of BocAP2/ERFs by RT-qPCR in Different Tissues

**Figure 10:**
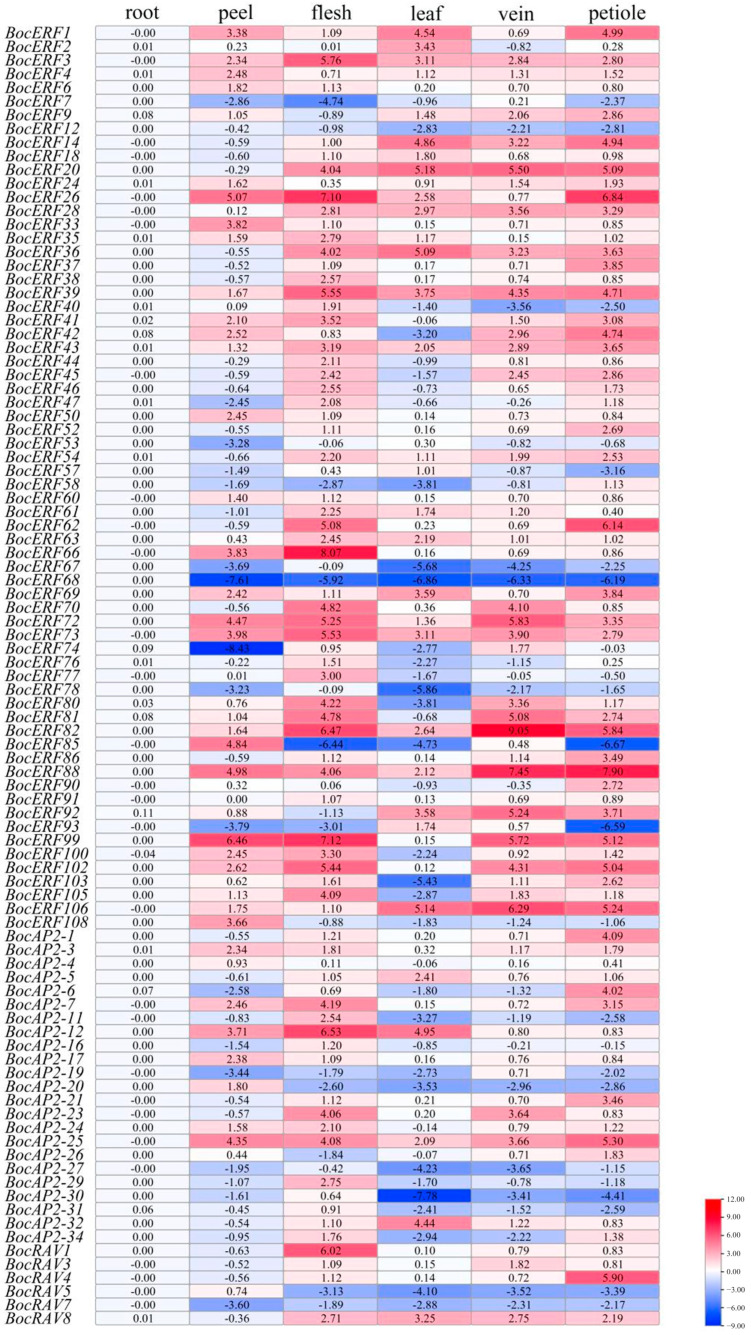
Reverse-transcription quantitative PCR analysis of ninety-five *BocAP2*/*ERF* transcription factors in roots, peels, flesh, leaves, veins, and petioles of kohlrabi. Normalized expression levels of *BocAP2*/*ERF* genes in different tissues. Data in the heatmap box are normalized expression levels of three replicates.

The relative expression levels of *BocAP2*/*ERF*s in different tissues in kohlrabi were obtained by qRT-PCR (Figure 10). The primers used are listed in Table S8. Ninety-five *BocAP2*/*ERF* genes showed relatively lower expression levels in roots. Two *BocAP2*/*ERF* genes, *BocERF26 and BocERF99*, showed higher expression levels in peels. Twelve *BocAP2*/*ERF* genes showed relatively higher expression levels in flesh, including *BocERF3*, *BocERF26*, *BocERF39*, *BocERF62*, *BocERF66*, *BocERF72*, *BocERF73*, *BocERF82*, *BocERF99*, *BocERF102*, *BocAP2-12* and *BocRAV1*. Three *BocAP2*/*ERF* genes showed relatively higher expression levels in leaves: *BocERF20*, *BocERF36* and *BocERF106*. Twelve *BocAP2*/*ERF* genes showed relative higherly expression levels in veins: *BocERF20*, *BocERF72*, *BocERF81*, *BocERF82*, *BocERF66*, *BocERF72*, *BocERF73*, *BocERF82*, *BocERF88*, *BocERF92*, *BocERF99* and *BocERF106*. Ten *BocAP2*/*ERF* genes showed relatively higher expression levels in petioles: *BocERF20*, *BocERF26*, *BocERF62*, *BocERF82*, *BocERF88*, *BocERF99*, *BocERF102*, *BocERF106*, *BocAP2-25* and *BocRAV4*. The expression of *BocAP2*/*ERF* family genes of kohlrabi have obvious specificity in different tissues that might be related to the function of specific organs.

## 3. Discussion

Plant response to stress is a complex process that involves the interaction of different stress-sensitive mechanisms [31]. In order to explore the regulatory mechanism of kohlrabi in response to osmotic stress, we measured the physiological indices associated with osmotic stress in plants, screened the DEGs based on transcriptomes, and subsequently identified and analyzed the *AP2*/*ERF* transcription factor family.

Previous studies showed that plants purge water deficit-induced excess ROS by developing a stronger antioxidative defense system using such enzymes as SOD and CAT, thus alleviating the destructive effects of water deficit on the plant [32,33]. Under conditions of water deficit, plants significantly increase their proline content and CAT activity compared with the normal environment, and SOD activity shows an overall upward trend [34]. In this study, the variation trends for proline content, SOD activity and CAT activity measured at different time points were basically consistent with the above results. Plants produce SOD, CAT and proline to regulate gene expression in response to water deficit [35]. Based on weighted gene correlation network analysis, multiple families of transcription factors had high degrees of interaction with proline content, superoxide dismutase activity and catalase activity, mainly including *AP2*/*ERF*s, *NAC*s and eight other transcription factor families.

The *AP2*/*ERF* transcription factor family is one of the largest transcription factor families in plants, and plays an important role in plant growth and response to stress [10,35,36,37]. Kong et al. [38] proved that overexpression of *PtoERF15* contributed to stem water potential maintenance in response to water deficit in *Populus tomentosa*, thus promoting drought tolerance. Similarly, Li et al. [39] revealed that overexpression of *AtruDREB28* increased tolerance to drought stress by enhancing reactive oxygen species-scavenging capability in *Acer truncatum*. Zhu et al. [40] reported that *CqERF24* overexpression in *Arabidopis thaliana* lines could enhance drought resistance through increased antioxidant enzyme activity and activated related stress genes, while silencing *CqERF24* in quinoa decreased drought tolerance and overexpression of *CqERF24* in quinoa calli enhanced resistance to mannitol. In this study, we undertook a transcriptome search for *AP2*/*ERF* family genes in kohlrabi and identified 151 *AP2*/*ERF* transcription factors. The specific expression analysis of *BocAP2*/*ERF*s in six different tissues showed that the expression of *BocAP2*/*ERF*s was higher in flesh and veins, but lower in roots. PEG-6000 osmotic stress-induced expression analysis of *BocAP2*/*ERF* family genes showed that 27 *BocAP2*/*ERF*s were induced by osmotic stress, of which 11 genes were upregulated varying degrees and 16 genes were downregulated. In addition, some *BocAP2*/*ERF* genes were obviously induced by osmotic stress in leaves, and the expression levels showed a trend of “up–down–up” in general, with high expression characteristics at 48 h under osmotic stress. According to the analysis of *BocAP2*/*ERF* expression characteristics, *BocAP2*/*ERF* genes are involved in plant response to abiotic stress, which may depend on the response site and the time and severity of the stress. Some *BocAP2*/*ERF* transcription factors showed tissue-specific expression, and many other candidate genes in the *BocAP2*/*ERF* family may also be involved in stress response and plant development.

## 4. Materials and Methods

### 4.1. Plant Materials and Sample Collection

The kohlrabi cultivated variety C8, provided by the *Brassica* vegetable crop breeding team at the Academy of Agriculture and Forestry Sciences of Qinghai University, was planted in a light incubator (Thermo Fisher Scientific, Waltham, MA, USA) under 12 h light/12 dark at 25 ± 1 °C, 65% relative humidity, and 120 mmol m^−2^ s^−1^ light intensity. After 40 days, growing seedlings were watered to saturation, and 24 h later, the control seedlings leaf samples (0 h) were collected and all seedlings were irrigated with 25% PEG-6000. Then, the osmotic stress leaf samples were collected at 12, 24 and 48 h later with three biological replicates for each treatment and each biological replicate containing seven individual plants. The collected leaves were immediately frozen and stored at −80 °C.

### 4.2. CAT and SOD Antioxidant Enzyme Activity and Proline Content Measurement

CAT activity, SOD activity and proline content were measured using a CAT activity detection kit (BC0205), a superoxide dismutase activity detection kit (BC0175) and proline content detection kit (BC0295), respectively. All detection kits were purchased from Beijing Solarbio Science and Technology Co., Ltd. (Beijing, China). The specific operation processes of the three indicators were followed according to the instructions of the kit.

### 4.3. RNA-Sequencing Data Assembly and Gene Annotation

Processes from total RNA extraction to gene annotation were conducted by Wuhan Metware Biotechnology Co. Ltd. (Wuhan, China) following the methods described by Lu et al. [41]. Based on the Trinity software 2.1.1, the short reads were assembled [42] and the assembled sequences were used as the reference. The unigene sequence was annotated using the BLAST searcher on KEGG, NR, Swiss-Prot, GO, KOGs, Trembl, and Pfam databases separately. The similarity between the transcript sequences of the species and similar species were compared by Nr database. The dataset can be searched from the NCBI Short Read Archive (SRA) under accession number PRJNA1051351, which contains 12 RNA-seq data from 12 libraries: SRR27183516, SRR27183515, SRR27183514, SRR27183507, SRR27183506, SRR27183505, SRR27183513, SRR27183512, SRR27183511, SRR27183510, SRR27183509 and SRR27183508.

### 4.4. Analysis and Screening of Differentially Expressed Genes under Osmotic Stress

To be able to accurately analyze the levels of differential expression in genes, clean reads of each sample were derived and mapped onto the reference by bowtie2 in RSEM software [43]. The number of mapped reads and transcript lengths were normalized using FPKM (fragments per kilobase of transcript per million fragments mapped). In the process of screening DEGs, we performed differential analyses between sample groups using the DESeq2 package [44]. To obtain the false-discovery rate (FDR), we performed strict hypothesis-testing correction for the hypothesis probability (*p*-value). The requirements for differential gene screening were |log_2_Folge| ≥ 1, with FDR < 0.05.

### 4.5. Validation of DEGs

To confirm the accuracy of the RNA-seq data of kohlrabi seedlings under PEG-6000 osmotic stress, eight DEGs were casually screened for RT-qPCR experiments. Three biological and three technical replicates were tested.

### 4.6. Weighted Gene Co-Expression Network Analysis

The co-expression network among FPKM values of DEGs and physicochemical parameters was generated using the weighted gene co-expression network analysis in R package 3.16.5 [45] based on Pearson’s correlation coefficient. The requirements for co-expression gene screening were |r| > 0.8, with *p* < 0.05. Cytoscape v3.8.0 [46] was used to visualize the co-expression network.

### 4.7. Identification of BocAP2/ERF Transcription Factor Family Members in Kohlrabi

According to the transcriptome unigene annotation file, AP2 and ERF as keywords were filtered in the KEGG, NR and Swiss-Prot annotation files, respectively. The CDS sequences searched were submitted to NCBI for online BLAST analysis, cluster and homologous sequences analyzed were aligned using DNAMAN 9.0 software, and the false-positive sequences were eliminated. Full-length CDS sequences of 151 *BocAP2*/*ERF* sequences were found. The predicted physicochemical parameters of BocAP2/ERF proteins were predicted with ProtParam. Motif prediction analysis was performed using the website tool MEME [47]. Tbtools software V1.098 [48] was used to visualize the conserved motifs and domains.

### 4.8. Phylogenetic Analysis and Chromosomal Distribution of BocAP2/ERF Transcription Factor Family

The AP2/ERF amino acid sequences of *A. thaliana* were downloaded from the Arabidopsis Information Resource (https://www.arabidopsis.org, accessed on 23 December 2023) database. The 287 amino acid sequences were aligned in MEGA 11 for the construction of the phylogenetic tree and 1000 bootstrap replicates [49,50]. The ultimate phylogenetic trees were visualized and beautified using Evolview 3.0 (www.evolgenius.info, accessed on 23 December 2023). The chromosomal localization information of the BocAP2/ERF genes in kohlrabi was retrieved from the *Brassica oleracea* genome database, and then analyzed using TBtools software V1.098 [48].

### 4.9. cDNA Synthesis and Expression Analysis by Quantitative Real-Time PCR

CDNA synthesis, final cDNA concentration adjustment, and RT-qPCR were performed following the manufacturer’s instructions as per Ren et al. [51]. The *TIP4* gene was used as an internal control. RT-qPCR specific primers were designed with Primer Premier 5 software. The relative expression levels of genes were measured using the 2^−ΔΔCt^ method [52]. The relative expression was analyzed using Excel 97-2003 and plotted heatmaps using TBtools software [48].

## 5. Conclusions

In this study, 282 DEGs were analyzed and screened under osmotic stress in kohlrabi by RNA-seq data. The *AP2*/*ERF* transcription factor family, *NAC* transcription factor family and eight other transcription factor families were identified, with high degrees of interaction with CAT and SOD antioxidant enzyme activity and proline content. Subsequently, 151 *AP2*/*ERF* genes were identified and analyzed. Twelve conserved motifs were searched and all *AP2*/*ERF* genes were clustered into four groups. A total of 149 *AP2*/*ERF* genes were randomly distributed on the chromosome, and relative expression level analysis showed that *BocAP2*/*ERF* genes of kohlrabi have obvious specificity in different tissues. According to the analysis of *BocAP2*/*ERF* expression characteristics, *BocAP2*/*ERF* genes are involved in plant response to abiotic stress, which may depend on the response site and the time and severity of the stress. This study lays a foundation for explaining the osmotic stress resistance mechanism of kohlrabi and provides a theoretical basis for functional analysis of BocAP2/ERF transcription factor family members.

## Figures and Tables

**Table 1:** Functional annotation of assembled unigenes under polyethylene glycol 6000 osmotic stress in kohlrabi seedling.

Database	Number of Unigenes	Percentages (%)
KEGG	134,011	68.15
NR	173,759	88.36
GO	144,868	73.67
KOG	103,662	52.72
Pfam	123,671	62.89
Swiss-Prot	125,704	63.93
Trembl	175,825	89.41
Annotated in at least one database	177,301	90.16
Total unigenes	196,642	100.00

## Data Availability

The dataset is available from the NCBI Short Read Archive (SRA) under accession number PRJNA1051351.

## Supplementary Materials

The following supporting information can be downloaded at https://www.mdpi.com/article/10.3390/plants13081167/s1. Table S1: Summary of kohlrabi seedling RNA-sequencing data under polyethylene glycol 6000 osmotic stress in this study. Table S2: All differentially expressed unigenes were identified in the pairwise comparison of 0 h vs. 12 h, 0 h vs. 24 h, 0 h vs. 48 h, 12 h vs. 24 h, 12 h vs. 48 h and 24 h vs. 48 h. Table S3: A total of 1541 common differentially expressed unigenes were identified in the pairwise comparison of treatment groups and control (0 h vs. 12 h, 0 h vs. 24 h and 0 h vs. 48 h). Table S4: Overall 684 common differentially expressed unigenes were identified in the pairwise comparison of 0 h vs. 12 h, 12 h vs. 24 h and 24 h vs. 48 h. Table S5: There were 2383 unigenes in subclass 7 with a downward trend and 2583 unigenes in subclass 8 with a upward trend. Table S6: Primers used for validation of the transcriptomic data by the qRT-PCR experiment. Table S7: Physicochemical property analysis of 151 *BocAP2*/*ERF*s in kohlrabi. Table S8: Primers used for the relative expression levels of BocAP2/ERFs in different tissues in kohlrabi. Figure S1: Transcriptome annotation of all unigenes in kohlrabi seedlings under PEG6000 osmotic stress. (a) Distribution of transcript and unigene sequence lengths. (b) Top BLAST hit species based on unigene annotation of kohlrabi leaves. Seven top hit species were identified.
