# Supplementary material for: Differentially Expressed Genes Identification of Kohlrabi Seedlings (Brassica oleracea var. caulorapa L.) under Polyethylene Glycol Osmotic Stress and AP2/ERF Transcription Factor Family Analysis
Source: Plants (Basel). 2024 Apr 22;13(8):1167. doi: 10.3390/plants13081167 (PMC11054715; doi:10.3390/plants13081167)
Supplement: Supplementary file 1 [file plants-13-01167-s001.zip › supplementary materials/Table S1.pdf]

Table S1. Summary of kohlrabi seedlings RNA-sequencing data under polyethylene glycol 6000-simulated drought stress in this study.

Sample	Raw Reads	Clean Reads	Clean Base(G)	Q20(%)	Q30(%)	GC Content(%)
CK-1	51073388	48270724	7.24	98.03	94.16	47.35
CK-2	47026420	44666012	6.70	98.12	94.36	47.30
CK-3	51183882	48709548	7.31	98.14	94.44	47.42
12h-1	45262254	42976838	6.45	98.40	95.12	47.10
12h-2	49158404	45739148	6.86	97.84	93.91	46.96
12h-3	48043564	45358166	6.80	98.00	94.11	47.09
24h-1	46365532	44650646	6.70	98.20	94.53	47.08
24h-2	51795110	50095338	7.51	98.28	94.75	47.27
24h-3	53167538	51045458	7.66	98.07	94.29	47.58
48h-1	46653846	43780842	6.57	98.16	94.60	46.38
48h-2	45548446	42914504	6.44	98.12	94.41	47.01
48h-3	53042244	50114348	7.52	98.03	94.14	47.26
