# Supplementary material for: Differentially Expressed Genes Identification of Kohlrabi Seedlings (Brassica oleracea var. caulorapa L.) under Polyethylene Glycol Osmotic Stress and AP2/ERF Transcription Factor Family Analysis
Source: Plants (Basel). 2024 Apr 22;13(8):1167. doi: 10.3390/plants13081167 (PMC11054715; doi:10.3390/plants13081167)
Supplement: Supplementary file 1 [file plants-13-01167-s001.zip › supplementary materials/Table S7.pdf]

**Table S7.** Physicochemical property analysis of 151 *BocAP2/ERFs* in kohlrabi.

| Gene ID | Sequence ID | Accession number of NCBI Reference Sequence | Length of cDNA | Number of amino acids (aa) | Molecular Weight (Da) | Theoretical pI | Total number of atoms | Aliphatic index | Grand average of hydropathicity |
|---|---|---|---|---|---|---|---|---|---|
| *BocERF1* | Cluster-17807.98657 | XM_013757078.1 | 1305 | 434 | 47518.81 | 8.50 | 6545 | 57.88 | -0.685 |
| *BocERF2* | Cluster-8561.0 | XM_013759052.1 | 1323 | 440 | 48877.67 | 8.67 | 6760 | 62.98 | -0.593 |
| *BocERF3* | Cluster-17807.113651 | XM_013763491.1 | 570 | 189 | 21216.39 | 5.89 | 2905 | 46.56 | -0.921 |
| *BocERF4* | Cluster-17807.67803 | XM_013774334.1 | 1062 | 353 | 39674.11 | 4.99 | 5476 | 58.67 | -0.778 |
| *BocERF5* | Cluster-17807.21654<br>Cluster-17807.108274<br>Cluster-17807.108275<br>Cluster-17807.15765 | XM_013775771.1 | 1221 | 406 | 45177.47 | 8.55 | 6283 | 62.78 | -0.787 |
| *BocERF6* | Cluster-17807.119527 | XM_013880612.3 | 819 | 272 | 30696.37 | 4.99 | 4252 | 69.26 | -0.521 |
| *BocERF7* | Cluster-17807.97937 | NM_001315928.1 | 669 | 222 | 24734.94 | 9.12 | 3483 | 70.32 | -0.652 |
| *BocERF8* | Cluster-17807.109356<br>Cluster-17807.47931 | NM_001316282.1 | 621 | 206 | 23288.72 | 4.94 | 3214 | 63.01 | -0.787 |
| *BocERF9* | Cluster-17807.108193 | XM_006297821.2 | 1158 | 385 | 43267.81 | 5.06 | 5938 | 50.73 | -0.846 |
| *BocERF10* | Cluster-17807.23530 | XM_009109191.2 | 468 | 155 | 17556.09 | 10.00 | 2476 | 72.52 | -0.646 |
| *BocERF11* | Cluster-13788.0<br>Cluster-17807.11695 | XM_009135261.3 | 1269 | 422 | 46694.43 | 8.80 | 6498 | 64.34 | -0.694 |
| *BocERF12* | Cluster-17807.111848 | XM_009148142.3 | 1140 | 379 | 41844.10 | 4.89 | 5725 | 49.50 | -0.793 |
| *BocERF13* | Cluster-17807.14842 | XM_013728592.1 | 735 | 244 | 27341.80 | 5.32 | 3828 | 72.38 | -0.631 |
| *BocERF14* | Cluster-17807.75233 | XM_013728852.1 | 567 | 188 | 20415.04 | 5.44 | 2836 | 69.10 | -0.408 |
| *BocERF15* | Cluster-17807.123753 | XM_013731560.1 | 1146 | 381 | 42105.37 | 4.98 | 5764 | 50.29 | -0.806 |
| *BocERF16* | Cluster-17807.67619 | XM_013731560.1 | 1131 | 376 | 41436.72 | 4.88 | 5669 | 48.62 | -0.757 |
| *BocERF17* | Cluster-17807.67620 | XM_013731560.1 | 1140 | 379 | 41777.10 | 4.98 | 5720 | 49.53 | -0.782 |
| *BocERF18* | Cluster-19609.0 | XM_013734904.1 | 768 | 255 | 28706.88 | 5.07 | 3972 | 58.59 | -0.850 |
| *BocERF19* | Cluster-26482.0 | XM_013734904.1 | 774 | 257 | 28861.05 | 5.20 | 3993 | 60.04 | -0.817 |
| *BocERF20* | Cluster-12843.0 | XM_013736990.1 | 552 | 183 | 20061.29 | 5.71 | 2739 | 57.16 | -0.502 |
| *BocERF21* | Cluster-17807.103870<br>Cluster-17807.28353 | XM_013737154.1 | 1005 | 334 | 36666.01 | 5.68 | 5095 | 68.20 | -0.494 |
| *BocERF22* | Cluster-17807.111305<br>Cluster-17807.119164 | XM_013738045.1 | 816 | 271 | 30222.04 | 5.06 | 4222 | 76.68 | -0.441 |
| *BocERF23* | Cluster-17807.111093 | XM_013738368.1 | 771 | 256 | 28556.77 | 5.75 | 3994 | 66.64 | -0.709 |
| *BocERF24* | Cluster-17807.45964<br>Cluster-17807.98579 | XM_013739850.1 | 561 | 186 | 21008.19 | 5.56 | 2903 | 58.28 | -0.996 |
| *BocERF25* | Cluster-20522.0 | XM_013741144.1 | 786 | 261 | 29773.79 | 5.10 | 4065 | 52.76 | -0.903 |

| | | | | | | | | | |
|---|---|---|---|---|---|---|---|---|---|
| *BocERF26* | Cluster-20681.0 | XM_013741329.1 | 480 | 159 | 17592.91 | 7.04 | 2444 | 68.74 | -0.715 |
| *BocERF27* | Cluster-17807.7585 | XM_013741497.1 | 456 | 151 | 16380.10 | 6.27 | 2234 | 65.89 | -0.415 |
| *BocERF28* | Cluster-17807.74068 | XM_013743782.1 | 576 | 191 | 21007.17 | 7.10 | 2883 | 59.42 | -0.820 |
| *BocERF29* | Cluster-17807.70200 | XM_013745251.1 | 489 | 162 | 18023.65 | 9.60 | 2530 | 71.79 | -0.511 |
| *BocERF30* | Cluster-15258.0 | XM_013746533.1 | 1035 | 344 | 38331.13 | 9.47 | 5348 | 67.79 | -0.585 |
| *BocERF31* | Cluster-17807.152397 | XM_013746533.1 | 1035 | 344 | 38398.19 | 9.53 | 5349 | 65.52 | -0.609 |
| *BocERF32* | Cluster-17807.132655<br>Cluster-17807.97987 | XM_013747492.1 | 597 | 198 | 21936.87 | 5.51 | 3047 | 68.08 | -0.475 |
| *BocERF33* | Cluster-17807.126676 | XM_013747936.1 | 687 | 228 | 25924.54 | 4.89 | 3556 | 56.93 | -0.711 |
| *BocERF34* | Cluster-17807.45997 | XM_013748703.1 | 732 | 243 | 27130.37 | 5.41 | 3783 | 67.45 | -0.696 |
| *BocERF35* | Cluster-17807.63901 | XM_013748703.1 | 732 | 243 | 27129.38 | 5.54 | 3784 | 67.45 | -0.696 |
| *BocERF36* | Cluster-17807.19103 | XM_013749655.1 | 540 | 179 | 19828.86 | 4.63 | 2701 | 51.28 | -0.570 |
| *BocERF37* | Cluster-17807.78121 | XM_013751691.1 | 663 | 220 | 24387.46 | 9.30 | 3420 | 70.09 | -0.665 |
| *BocERF38* | Cluster-17807.145380<br>Cluster-17807.147440 | XM_013753923.1 | 864 | 287 | 31479.61 | 8.56 | 4431 | 79.97 | -0.403 |
| *BocERF39* | Cluster-17807.112787 | XM_013755124.1 | 1092 | 363 | 40562.85 | 4.92 | 5573 | 53.28 | -0.805 |
| *BocERF40* | Cluster-17807.72481 | XM_013755124.1 | 1092 | 363 | 40530.79 | 4.92 | 5572 | 54.08 | -0.799 |
| *BocERF41* | Cluster-17807.136204 | XM_013755131.1 | 1065 | 354 | 39472.76 | 5.03 | 5435 | 53.79 | -0.767 |
| *BocERF42* | Cluster-17807.56242 | XM_013755131.1 | 1053 | 350 | 39335.55 | 5.32 | 5404 | 51.06 | -0.783 |
| *BocERF43* | Cluster-17807.58663 | XM_013755131.1 | 1062 | 353 | 39399.77 | 5.09 | 5429 | 54.79 | -0.759 |
| *BocERF44* | Cluster-17807.56240 | XM_013755131.1 | 1062 | 353 | 39353.68 | 5.09 | 5425 | 55.61 | -0.753 |
| *BocERF45* | Cluster-17807.56241 | XM_013755131.1 | 1062 | 353 | 39367.71 | 5.09 | 5428 | 55.61 | -0.753 |
| *BocERF46* | Cluster-17807.134025<br>Cluster-17807.59127 | XM_013755131.1 | 1062 | 353 | 39309.65 | 5.08 | 5416 | 54.79 | -0.758 |
| *BocERF47* | Cluster-17807.84610 | XM_013755972.1 | 540 | 179 | 20250.39 | 5.83 | 2799 | 56.15 | -0.980 |
| *BocERF48* | Cluster-17807.90768 | XM_013756535.1 | 768 | 255 | 28554.81 | 7.73 | 3958 | 62.04 | -0.780 |
| *BocERF49* | Cluster-17807.116927 | XM_013757077.1 | 1365 | 454 | 49647.24 | 8.21 | 6847 | 60.90 | -0.649 |
| *BocERF50* | Cluster-17807.116928 | XM_013757077.1 | 1365 | 454 | 49674.35 | 8.21 | 6858 | 62.38 | -0.627 |
| *BocERF51* | Cluster-17807.43103<br>Cluster-17807.116925 | XM_013757077.1 | 1365 | 454 | 49656.25 | 8.21 | 6847 | 60.90 | -0.648 |
| *BocERF52* | Cluster-17807.116926 | XM_013757077.1 | 1365 | 454 | 49561.19 | 8.21 | 6842 | 62.18 | -0.627 |
| *BocERF53* | Cluster-17807.104480 | XM_013757078.1 | 1305 | 434 | 47518.81 | 8.50 | 6545 | 57.88 | -0.685 |
| *BocERF54* | Cluster-17807.116929<br>Cluster-17807.155014 | XM_013757078.1 | 1305 | 434 | 47489.86 | 8.71 | 6548 | 59.22 | -0.656 |
| *BocERF55* | Cluster-17807.140788 | XM_013759052.1 | 1323 | 440 | 48763.52 | 8.47 | 6745 | 63.43 | -0.590 |
| *BocERF56* | Cluster-17807.19661 | XM_013759052.1 | 1323 | 440 | 48793.55 | 8.47 | 6749 | 63.20 | -0.596 |

| | | | | | | | | | |
|---|---|---|---|---|---|---|---|---|---|
| *BocERF57* | Cluster-17807.3481 | XM_013759052.1 | 1323 | 440 | 48745.49 | 8.47 | 6747 | 64.32 | -0.584 |
| *BocERF58* | Cluster-17807.68685 | XM_013759052.1 | 1323 | 440 | 48877.67 | 8.67 | 6760 | 62.98 | -0.593 |
| *BocERF59* | Cluster-17807.142693 | XM_013759059.1 | 1287 | 428 | 47144.87 | 8.86 | 6543 | 64.74 | -0.562 |
| *BocERF60* | Cluster-17807.55305 | XM_013759059.1 | 1287 | 428 | 47172.85 | 8.70 | 6536 | 64.30 | -0.559 |
| *BocERF61* | Cluster-17807.68686 | XM_013759059.1 | 1287 | 428 | 47135.83 | 8.71 | 6534 | 63.83 | -0.565 |
| *BocERF62* | Cluster-17807.33829 | XM_013760691.1 | 465 | 154 | 17127.09 | 6.42 | 2369 | 66.62 | -0.515 |
| *BocERF63* | Cluster-17807.67932 | XM_013760154.1 | 933 | 310 | 34420.74 | 8.70 | 4797 | 66.48 | -0.541 |
| *BocERF64* | Cluster-17807.149896<br>Cluster-17807.149892<br>Cluster-17807.149893 | XM_013766827.1 | 1284 | 427 | 46899.36 | 8.83 | 6518 | 61.10 | -0.757 |
| *BocERF65* | Cluster-17807.149895 | XM_013766827.1 | 1278 | 425 | 46979.65 | 8.79 | 6537 | 62.73 | -0.725 |
| *BocERF66* | Cluster-17807.150914 | XM_013766827.1 | 1284 | 427 | 46872.34 | 8.83 | 6515 | 61.10 | -0.750 |
| *BocERF67* | Cluster-17807.164 | XM_013766873.1 | 762 | 253 | 28235.72 | 5.23 | 3927 | 65.61 | -0.667 |
| *BocERF68* | Cluster-17807.40114 | XM_013766873.1 | 762 | 253 | 28204.74 | 5.00 | 3928 | 69.84 | -0.581 |
| *BocERF69* | Cluster-17807.119526 | XM_013770131.1 | 810 | 269 | 30408.05 | 5.17 | 4220 | 68.96 | -0.575 |
| *BocERF70* | Cluster-17807.148103 | XM_013770131.1 | 819 | 272 | 30722.50 | 5.17 | 4264 | 67.13 | -0.521 |
| *BocERF71* | Cluster-17807.145709 | XM_013770553.1 | 831 | 276 | 30091.12 | 5.52 | 4151 | 61.92 | -0.572 |
| *BocERF72* | Cluster-17807.130167 | XM_013774334.1 | 1047 | 348 | 39117.47 | 4.94 | 5391 | 58.39 | -0.778 |
| *BocERF73* | Cluster-17807.90406 | XM_013774335.1 | 1038 | 345 | 38859.24 | 4.94 | 5359 | 58.90 | -0.771 |
| *BocERF74* | Cluster-17807.102609<br>Cluster-17807.63424 | XM_013774807.1 | 954 | 317 | 35290.94 | 5.09 | 4837 | 50.88 | -0.734 |
| *BocERF75* | Cluster-17807.107222 | XM_013774807.1 | 954 | 317 | 35306.94 | 5.09 | 4838 | 50.57 | -0.742 |
| *BocERF76* | Cluster-17807.108276 | XM_013775770.1 | 1236 | 411 | 45798.17 | 8.69 | 6368 | 62.02 | -0.790 |
| *BocERF77* | Cluster-17807.19575 | XM_013775770.1 | 1254 | 417 | 46186.64 | 8.69 | 6427 | 63.45 | -0.762 |
| *BocERF78* | Cluster-17807.108277<br>Cluster-17807.108279 | XM_013775771.1 | 1221 | 406 | 45170.43 | 8.55 | 6278 | 61.82 | -0.783 |
| *BocERF79* | Cluster-11428.0 | XM_013782826.1 | 831 | 276 | 31207.79 | 4.95 | 4320 | 69.31 | -0.611 |
| *BocERF80* | Cluster-17807.105515 | XM_013783142.1 | 693 | 230 | 25485.05 | 4.94 | 3513 | 61.91 | -0.680 |
| *BocERF81* | Cluster-17807.132959 | XM_013782268.1 | 783 | 260 | 29495.06 | 5.59 | 4100 | 61.92 | -0.690 |
| *BocERF82* | Cluster-17807.159090 | XM_013783150.1 | 660 | 219 | 24321.89 | 4.90 | 3358 | 64.57 | -0.618 |
| *BocERF83* | Cluster-621.0 | XM_013779125.1 | 651 | 216 | 24431.63 | 8.67 | 3395 | 59.63 | -0.870 |
| *BocERF84* | Cluster-17807.77102<br>Cluster-17807.80319 | XM_013794541.3 | 987 | 328 | 36210.40 | 6.07 | 5005 | 59.30 | -0.566 |
| *BocERF85* | Cluster-17807.82175 | XM_013794541.3 | 966 | 321 | 35449.55 | 6.00 | 4899 | 59.38 | -0.555 |
| *BocERF86* | Cluster-12737.0 | XM_013814510.3 | 882 | 293 | 33222.08 | 6.46 | 4573 | 51.98 | -0.808 |
| *BocERF87* | Cluster-17807.4752 | XM_013800927.3 | 834 | 277 | 31680.29 | 6.61 | 4357 | 51.44 | -0.886 |

| | | | | | | | | | |
|---|---|---|---|---|---|---|---|---|---|
| *BocERF88* | Cluster-22256.0 | XM_013799429.3 | 693 | 230 | 25680.45 | 4.62 | 3545 | 65.74 | -0.548 |
| *BocERF89* | Cluster-23151.0 | XM_013814459.3 | 741 | 246 | 27714.1 | 5.9 | 3877 | 75.77 | -0.633 |
| *BocERF90* | Cluster-7744.0 | XM_013799772.3 | 573 | 190 | 20673.15 | 4.97 | 2833 | 57.68 | -0.501 |
| *BocERF91* | Cluster-17807.7192 | XM_013826556.3 | 789 | 262 | 29910.41 | 7.23 | 4141 | 66.34 | -0.826 |
| *BocERF92* | Cluster-17807.115520 | XM_013837776.3 | 771 | 256 | 28588.72 | 5.43 | 3989 | 66.64 | -0.685 |
| *BocERF93* | Cluster-17807.48648 | XM_013842689.3 | 783 | 260 | 29416.93 | 5.59 | 4093 | 63.42 | -0.694 |
| *BocERF94* | Cluster-17807.6879 | XM_013850443.3 | 966 | 321 | 35696.26 | 5.37 | 4886 | 53.58 | -0.693 |
| *BocERF95* | Cluster-17807.7524 | XM_013849742.3 | 687 | 228 | 25187.98 | 4.69 | 3474 | 65.88 | -0.431 |
| *BocERF96* | Cluster-17807.43104 | XM_013859430.3 | 1299 | 432 | 47500.02 | 8.71 | 6563 | 62.18 | -0.614 |
| *BocERF97* | Cluster-17807.97897 | XM_013859430.3 | 1299 | 432 | 47512.13 | 8.87 | 6570 | 63.08 | -0.6 |
| *BocERF98* | Cluster-17807.32362<br>Cluster-17807.148102 | XM_013880612.3 | 831 | 276 | 31201.74 | 4.95 | 4314 | 67.9 | -0.603 |
| *BocERF99* | Cluster-17807.26909 | XM_013880959.3 | 1143 | 380 | 41846.12 | 4.94 | 5727 | 49.13 | -0.798 |
| *BocERF100* | Cluster-17807.73973<br>Cluster-17807.86378 | XM_013880959.3 | 1143 | 380 | 41913.3 | 4.98 | 5745 | 50.92 | -0.773 |
| *BocERF101* | Cluster-17807.158542 | XM_013881405.3 | 1035 | 344 | 38257.94 | 9.41 | 5328 | 66.37 | -0.601 |
| *BocERF102* | Cluster-17807.154886<br>Cluster-17807.28766<br>Cluster-17807.28767<br>Cluster-17807.3601<br>Cluster-17807.9257 | XM_018637720.2 | 987 | 328 | 35960.77 | 5.59 | 4959 | 58.11 | -0.658 |
| *BocERF103* | Cluster-17807.7525 | XM_033286625.1 | 708 | 235 | 25936.45 | 4.65 | 3575 | 61.4 | -0.57 |
| *BocERF104* | Cluster-17807.25925 | XM_048770003.1 | 933 | 310 | 33777.88 | 7.8 | 4725 | 76.35 | -0.4 |
| *BocERF105* | Cluster-17807.147387<br>Cluster-17807.147397 | XM_048775894.1 | 1224 | 407 | 44685.89 | 5.7 | 6108 | 55.9 | -0.711 |
| *BocERF106* | Cluster-17807.154001<br>Cluster-17807.32361 | XM_048777716.1 | 831 | 269 | 30064.83 | 5.28 | 4182 | 68.92 | -0.518 |
| *BocERF107* | Cluster-17807.27028 | XM_048777716.1 | 831 | 269 | 30147.91 | 5.28 | 4195 | 68.92 | -0.531 |
| *BocERF108* | Cluster-17807.126599<br>Cluster-17807.32997 | XM_048778063.1 | 588 | 197 | 21881.98 | 6.55 | 2996 | 57.11 | -0.959 |
| *BocAP2-1* | Cluster-17807.147385 | XM_013767819.1 | 1245 | 414 | 45173.61 | 6.22 | 6181 | 55.22 | -0.67 |
| *BocAP2-2* | Cluster-17807.147389 | XM_013878360.3 | 1254 | 417 | 45598.91 | 6.61 | 6225 | 50.14 | -0.75 |
| *BocAP2-3* | Cluster-17807.84134<br>Cluster-17807.62454 | XM_009105449.3 | 1365 | 454 | 49530.13 | 6.48 | 6758 | 50.35 | -0.782 |
| *BocAP2-4* | Cluster-17807.62453 | XM_009105450.3 | 1320 | 439 | 47816.23 | 7.09 | 6520 | 48.52 | -0.817 |
| *BocAP2-5* | Cluster-17807.138105 | XM_013732410.1 | 927 | 308 | 35001.58 | 7.69 | 4826 | 53.31 | -0.952 |

| | | | | | | | | | |
|---|---|---|---|---|---|---|---|---|---|
| *BocAP2-6* | Cluster-17807.21178 | XM_013732410.1 | 927 | 308 | 35041.69 | 7.69 | 4837 | 54.9 | -0.93 |
| *BocAP2-7* | Cluster-17807.134397 | XM_013737986.1 | 1680 | 559 | 61778.02 | 6.84 | 8432 | 56.21 | -0.703 |
| *BocAP2-8* | Cluster-17807.134400 | XM_013737986.1 | 1680 | 559 | 61876.08 | 6.82 | 8441 | 55.51 | -0.72 |
| *BocAP2-9* | Cluster-17807.157237<br>Cluster-17807.102797<br>Cluster-17807.107776<br>Cluster-17807.132097<br>Cluster-17807.102796<br>Cluster-17807.30266<br>Cluster-17807.30267<br>Cluster-17807.58977<br>Cluster-17807.13200 | XM_013738114.1 | 1086 | 361 | 40835.46 | 9.11 | 5632 | 55.12 | -0.831 |
| *BocAP2-10* | Cluster-11338.0 | XM_013751793.1 | 1767 | 588 | 65562.77 | 6.22 | 8957 | 59.18 | -0.773 |
| *BocAP2-11* | Cluster-17807.11162<br>Cluster-17807.36325 | XM_013763251.1 | 1668 | 555 | 61816.26 | 7.82 | 8462 | 56.59 | -0.7 |
| *BocAP2-12* | Cluster-28007.0 | XM_013763251.1 | 1668 | 555 | 61802.19 | 7.44 | 8455 | 56.59 | -0.699 |
| *BocAP2-13* | Cluster-17807.147394 | XM_013767819.1 | 1239 | 412 | 45084.42 | 6.43 | 6159 | 50.75 | -0.728 |
| *BocAP2-14* | Cluster-25753.0 | XM_013767843.1 | 1704 | 567 | 63688.79 | 6.29 | 8696 | 56.01 | -0.817 |
| *BocAP2-15* | Cluster-25753.1 | XM_013767843.1 | 1713 | 570 | 63967.23 | 6.47 | 8743 | 56.58 | -0.789 |
| *BocAP2-16* | Cluster-25753.4 | XM_013767843.1 | 1704 | 567 | 63726.8 | 6.24 | 8697 | 56.37 | -0.814 |
| *BocAP2-17* | Cluster-17807.110560 | XM_013783234.1 | 1014 | 337 | 38162.08 | 6.48 | 5255 | 55.34 | -0.958 |
| *BocAP2-18* | Cluster-17807.91274 | XM_013775779.1 | 1320 | 439 | 47652.97 | 6.73 | 6502 | 49.64 | -0.799 |
| *BocAP2-19* | Cluster-8935.0 | XM_013823335.3 | 1701 | 566 | 63609.9 | 6.47 | 8701 | 57.31 | -0.795 |
| *BocAP2-20* | Cluster-11675.0<br>Cluster-17807.55661 | XM_013833245.3 | 1014 | 337 | 38162.08 | 6.48 | 5255 | 55.34 | -0.958 |
| *BocAP2-21* | Cluster-17807.147390 | XM_013878360.3 | 1296 | 431 | 47272.81 | 6.08 | 6461 | 53.25 | -0.719 |
| *BocAP2-22* | Cluster-17807.147392<br>Cluster-17807.147395 | XM_013878360.3 | 1254 | 417 | 45598.91 | 6.61 | 6225 | 50.14 | -0.75 |
| *BocAP2-23* | Cluster-17807.147400 | XM_013878360.3 | 1254 | 417 | 45584.89 | 6.61 | 6222 | 49.9 | -0.749 |
| *BocAP2-24* | Cluster-17807.147402 | XM_013878360.3 | 1254 | 417 | 45583.98 | 6.61 | 6231 | 51.08 | -0.731 |
| *BocAP2-25* | Cluster-17807.147412<br>Cluster-17807.147398 | XM_013878360.3 | 1254 | 417 | 45320.72 | 6.14 | 6199 | 54.12 | -0.676 |
| *BocAP2-26* | Cluster-17807.147411<br>Cluster-17807.157563<br>Cluster-5730.0 | XM_013878360.3 | 1254 | 417 | 45612.98 | 6.84 | 6232 | 50.14 | -0.751 |
| *BocAP2-27* | Cluster-23389.0 | XM_018590187.2 | 1692 | 563 | 63087.26 | 6.12 | 8637 | 59.88 | -0.753 |

| | | | | | | | | | |
|---|---|---|---|---|---|---|---|---|---|
| *BocAP2-28* | Cluster-17807.37117 | XM_019237897.1 | 732 | 243 | 27238.56 | 4.78 | 3712 | 57.9 | -0.813 |
| *BocAP2-29* | Cluster-17807.134396 | XM_022700009.2 | 1674 | 557 | 62008.39 | 7.83 | 8496 | 57.58 | -0.738 |
| *BocAP2-30* | Cluster-17807.134401 | XM_022700009.2 | 1674 | 557 | 62020.64 | 7.45 | 8510 | 58.64 | -0.682 |
| *BocAP2-31* | Cluster-17807.134404 | XM_022700009.2 | 1674 | 557 | 61939.41 | 7.82 | 8496 | 59.16 | -0.712 |
| *BocAP2-32* | Cluster-17807.134407 | XM_022700009.2 | 1674 | 557 | 61969.44 | 7.82 | 8500 | 59.16 | -0.712 |
| *BocAP2-33* | Cluster-17807.21179 | XM_048761637.1 | 918 | 305 | 34666.22 | 7.69 | 4781 | 53.21 | -0.961 |
| *BocAP2-34* | Cluster-17807.147396 | XM_048775893.1 | 1296 | 431 | 47182.78 | 6.43 | 6456 | 53.92 | -0.72 |
| *BocRAV1* | Cluster-17807.82390 | XM_013782305.1 | 1026 | 341 | 38098.51 | 8.95 | 5303 | 67.36 | -0.628 |
| *BocRAV2* | Cluster-17807.49649 | XM_013744055.1 | 1023 | 340 | 38033.54 | 9.46 | 5311 | 70.68 | -0.6 |
| *BocRAV3* | Cluster-17807.64403 | XM_013744055.1 | 1014 | 337 | 38007.62 | 9.04 | 5292 | 69.02 | -0.642 |
| *BocRAV4* | Cluster-17807.132270 | XM_013763661.1 | 1032 | 343 | 38415.43 | 9.53 | 5389 | 74.08 | -0.475 |
| *BocRAV5* | Cluster-17807.918 | XM_013763730.1 | 1050 | 349 | 39109.04 | 9.31 | 5484 | 69.46 | -0.628 |
| *BocRAV6* | Cluster-17807.107998 | XM_013766842.1 | 1059 | 352 | 39460.37 | 9.36 | 5527 | 69.74 | -0.654 |
| *BocRAV7* | Cluster-17807.36555 | XM_013766842.1 | 1059 | 352 | 39529.56 | 9.24 | 5533 | 68.64 | -0.657 |
| *BocRAV8* | Cluster-17807.72819 | XM_013782456.1 | 1116 | 371 | 40869.18 | 9.41 | 5712 | 65.96 | -0.568 |
| *BocRAV9* | Cluster-17807.87240 | XM_013789359.3 | 1035 | 344 | 38620.36 | 9.15 | 5394 | 69.13 | -0.647 |
