# Supplementary figures and images for: Differentially Expressed Genes Identification of Kohlrabi Seedlings (Brassica oleracea var. caulorapa L.) under Polyethylene Glycol Osmotic Stress and AP2/ERF Transcription Factor Family Analysis
Source: Plants (Basel). 2024 Apr 22;13(8):1167. doi: 10.3390/plants13081167 (PMC11054715; doi:10.3390/plants13081167)

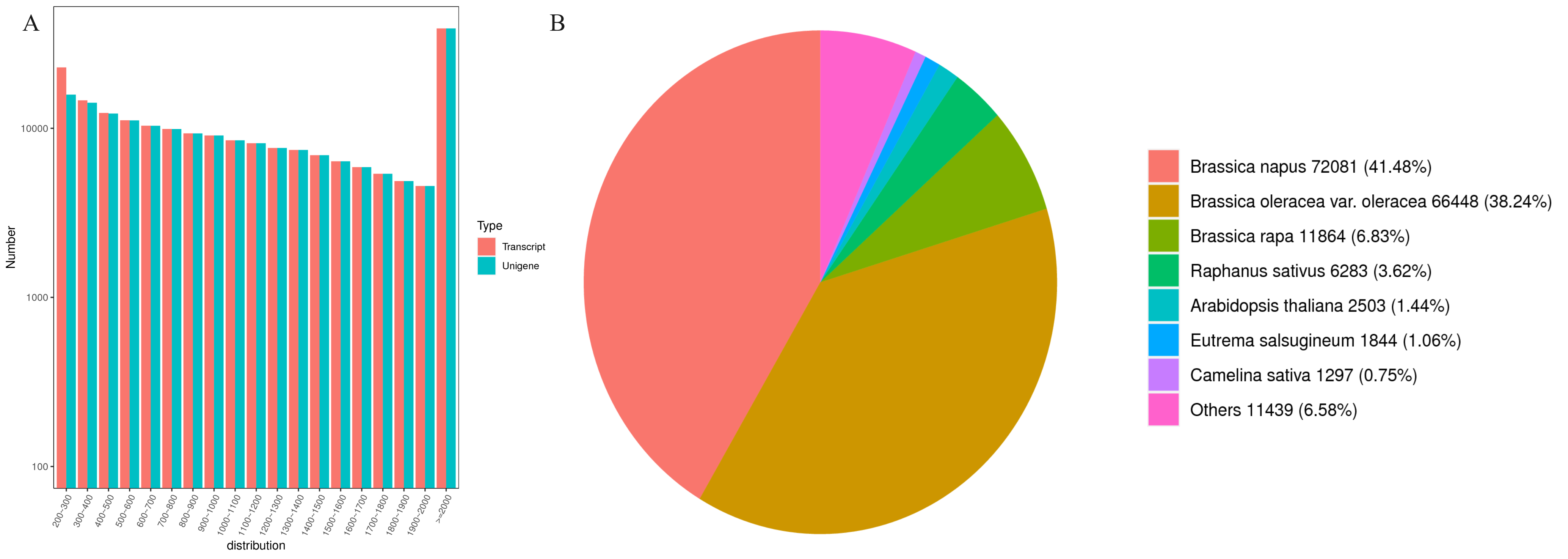

Supplement: Supplementary file 1 [file plants-13-01167-s001.zip › supplementary materials/figure S1.png]
